# Supplementary material for: Transglutaminase 2 Regulates HSF1 Gene Expression in the Acute Phase of Fish Optic Nerve Regeneration
Source: Int J Mol Sci. 2024 Aug 21;25(16):9078. doi: 10.3390/ijms25169078 (PMC11354351; doi:10.3390/ijms25169078)
Supplement: Supplementary file 1 [file ijms-25-09078-s001.zip › ijms-3105568-supplementary.pdf]

Figure S1

(a)

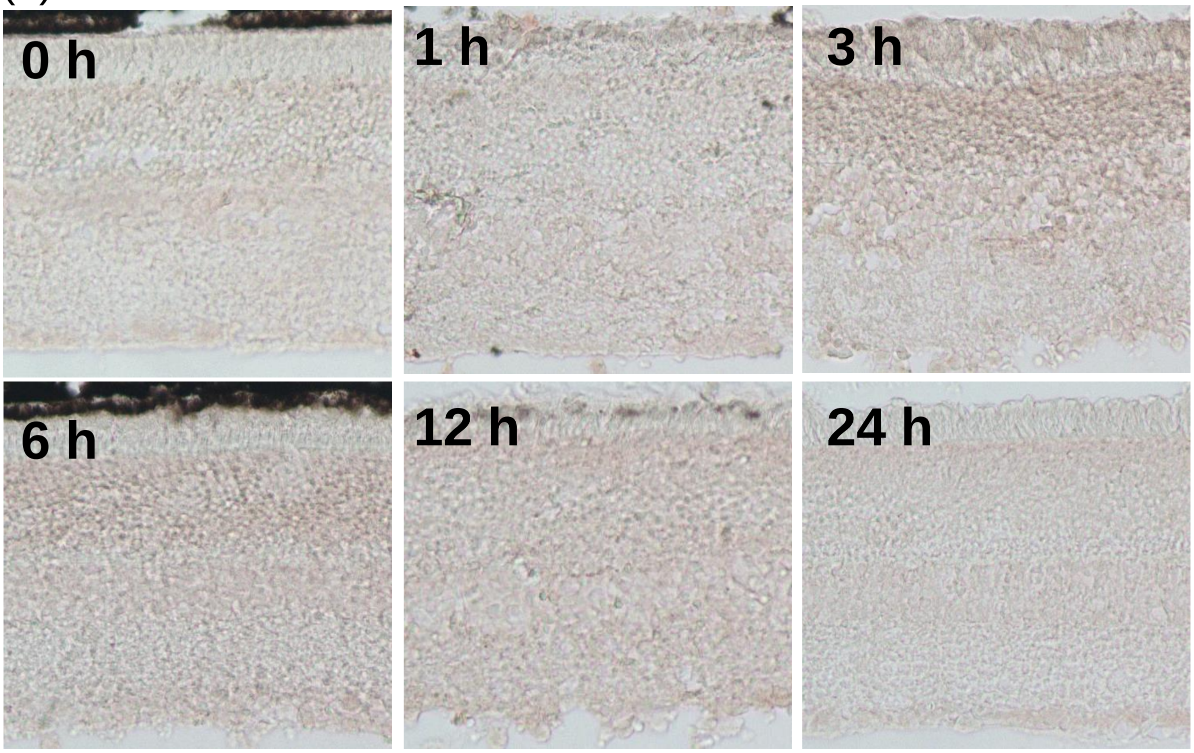

(b)

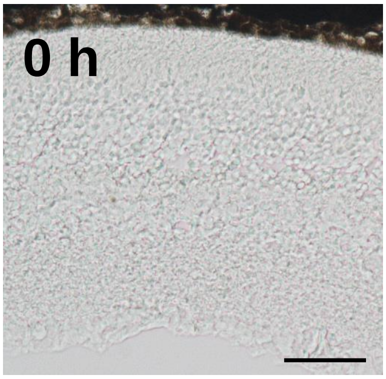

Figure S1.
(a) Immunohistochemical staining of HSF1 in the mouse retina after optic nerve lesions. In the mouse optic nerve crush model, almost no change in HSF1 expression was observed in the retina at each time point within 24 hours after optic nerve crush. **(b)** Negative control (no primary antibody control) of Immunohistochemical staining of HSF1 in the mouse retina. Scale bar, 50µm.

Figure S2

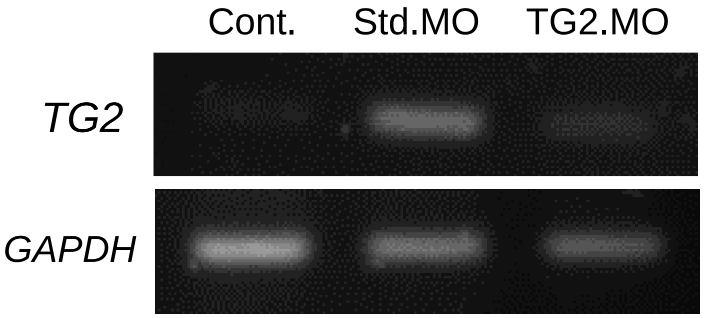

Figure S2

RT-PCR using real-time PCR primers at TG2 morphants (TG2.MO) showed low expression of TG2b than standard (Std) MO treated samples. Control (Cont.) sample is intact sample of optic nerve. TG2 expression is normally increased 0.5 h after optic nerve lesions as in the Std-MO group, but TG2b expression is markedly suppressed in TG2 morphants.
